# Botulinum Toxin for the Treatment of Hand Tremor

**DOI:** 10.3390/toxins10070299

**Published:** 2018-07-19

**Authors:** Nicki Niemann, Joseph Jankovic

**Affiliations:** Parkinson’s Disease Center and Movement Disorders Clinic, Department of Neurology, Baylor College of Medicine, Houston, TX 77030, USA; niemann@bcm.edu

**Keywords:** hand tremor, botulinum toxin, treatment, electromyography, kinematics, essential tremor, Parkinson’s disease, dystonic tremor

## Abstract

The aim of this study is to review our longitudinal experience with onabotulinumtoxinA (onaBoNT-A) injections for medically refractory hand tremor. We performed a retrospective review of our database of patients treated with onaBoNT-A for hand tremor evaluated between 2010 and 2018 in at least 2 sessions with follow-up. The majority were injected into the forearm flexors (FF), although treatment was individualized. During the specified period, 91 patients (53 essential tremor, 31 dystonic tremor, 6 Parkinson’s disease tremor, and 1 cerebellar outflow tremor) met our inclusion criteria. The mean age (SD) was 64.8 years (12.8), and mean duration of follow-up was 29.6 months (25.1) with mean of 7.7 (6.3) treatment visits. FF were injected in 89 (97.8%) patients, exclusively in 74 (81.3%), and 15 (16.5%) were injected in FF and other muscles. EMG guidance was used in 5 patients (5.5%). On a 0–4 “peak effect” rating scale (0 = no effect, 4 = marked improvement in severity and function), 80.2% and 85.7% of patients reported moderate or marked improvement (score 3 or 4) at their first and last follow-up visit, respectively. There was no statistically significant difference in the outcomes between first and last visit: average “peak effect” rating score (3.2 versus 3.4), “global” rating score (3.0 versus 3.2), latency of response (4.5 versus 3.8 days), and total duration of response (12.7 versus 12.8 weeks), except onaBoNT-A dose (65.0 versus 78.6 U/limb, *p* = 0.002). Of 1095 limb injections, there were 134 (12.2%) non-disabling and transient (mean 36 days) adverse events (132 limb weakness, 2 pain). OnaBoNT-A injections are safe and effective in the treatment of hand tremor.

## 1. Introduction

Tremor is defined as an involuntary, rhythmic, oscillatory movement of a body part [1]. Hand tremor is a common movement disorder, often associated with impairment in quality of life [2]. Essential tremor (ET) is the most common cause of postural and kinetic hand tremor; 4.6% of individuals 65 years or older are thought to have ET [3]. Parkinson’s disease (PD) affects 0.43–1.90% of individuals 60 years or older [4] with the majority experiencing rest or postural tremor [5,6]. While rest tremor is typically associated with PD, most PD patients also have a postural or re-emergent tremor, which is the most disabling form of PD-related tremor [6,7]. The prevalence of dystonic tremor is unknown [8], although hand tremor has been reported in up to 70% of patients in a series of patients with dystonia of various types [9]. There are many other causes of tremor besides ET, PD, and dystonia, which can affect not only hands but also other body parts [1]. Treatment of tremor with oral medications often provides insufficient relief, especially when the tremor is severe, and is frequently associated with systemic side effects [10,11]. Deep brain stimulation (DBS) is a highly effective treatment for tremor in ET and PD, but is invasive and can be associated with stimulation-induced and long-term side effects [12,13]. Functional lesional neurosurgery, especially focused ultrasound, has garnered more interest recently in the treatment of tremor but this intervention is not readily available and is associated with potentially serious adverse effects, particularly when performed bilaterally [14,15,16]. 

Botulinum toxin (BoNT) has been increasingly used in the treatment of focal tremors with reported good outcomes and long-term safety [17,18]. One of the main advantages of this treatment over above-noted strategies is the lack of systemic side effects. The aim of the present study is to describe our long-term experience with onabotulinumtoxinA (onaBoNT-A) in the treatment of medically refractory hand tremor using an individualized approach.

## 2. Results

**Figure 1:**
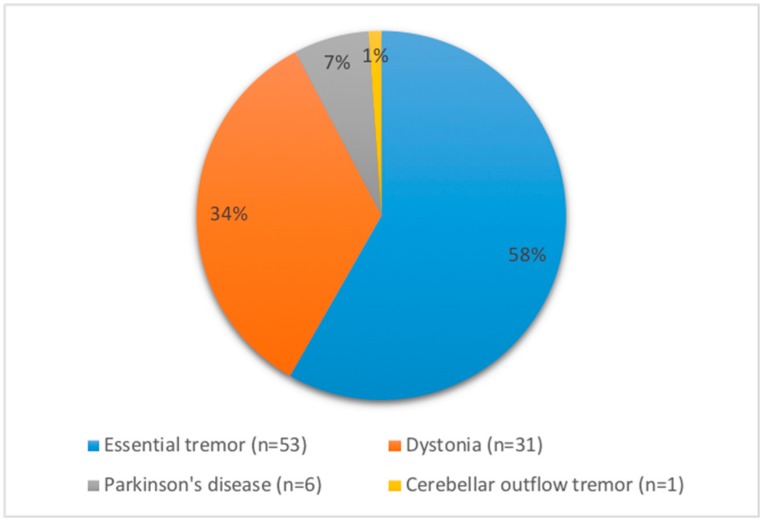
Etiology of hand tremor in patients treated with onabotulinumtoxinA (onaBoNT-A) injections from 1st January 2010 to 1st January 2018.

We identified a total of 91 patients who were treated with onaBoNT-A for hand tremor at least twice and had adequate follow-up during the period from 1st January 2010 to 1st January 2018. Of these patients, 23 (25.3%) received their initial injection prior to 1st January 2010. Diagnoses included ET (53 patients with 395 visits), PD (6 patients with 27 visits), dystonia (31 patients with 269 visits), and cerebellar outflow tremor (1 patient with 13 visits) (Figure 1). The male to female ratio was 1.1:1 (48 male and 43 female) and the mean age of all participants was 64.8 years at the time of their initial injection visit (Table 1). The mean duration of symptoms prior to the first onaBoNT-A injection was 23.2 years. The mean duration of follow-up from the first to the last injection visit was 29.6 months during which time patients underwent a mean of 7.7 injections. 

Forearm flexors were injected in 89 (97.8%) patients, 74 (81.3%) received injections exclusively to the forearm flexors, and 15 (16.5%) received injections to the forearm flexors and at least one other upper extremity muscle compartment (hand muscles, forearm extensors, arm flexors, arm extensors, and/or shoulder abductors); 2 (2.2%) received no forearm flexor injections. The specific muscles injected according to tremor etiology are listed in Table 2. EMG was used in 5 (5.5%) patients while the rest were injected using surface anatomy. The mean dose of onaBoNT-A increased significantly from 65.0 to 78.6 (*p* = 0.002) between the first and last visit; the mean dose was 71.8 U per limb. There was no statistically significant difference between outcomes from the first and last visit with respect to all other outcome measures: peak effect score was 3.2 versus 3.4 (*p* = 0.17), global rating was 3.0 versus 3.2 (*p* = 0.06), and latency of response was 4.5 versus 3.8 days (*p* = 0.10) (Table 3). Using the peak effect rating scale, 80.2% and 85.7% of patients rated their improvement as either moderate or marked (score of 3 or 4) at their first and last visit, compared to 74.7% and 80.2% of patients on the global effect rating scale at the first and last visit, respectively. A separate analysis comparing outcomes on the global rating and peak effect rating scale within each group (e.g., ET outcomes after first versus last injection) and between groups (e.g., ET versus dystonia) did not reveal any statistically significant differences (data not shown). The mean duration of maximum response at the first and last injection was 12.1 and 12.7 weeks (*p* = 0.70), respectively. The mean total duration of response at the first and last injection was 12.7 and 12.8 weeks (*p* = 0.87), respectively, but this is likely an underestimate as the effects of the prior injection had not fully worn off in 27 (29.7%) and 41 (45.1%) patients at the time of follow-up after the first and last visit. Of the total 1095 limbs injected, 134 (12.2%) were associated with some adverse effect (120 hand grip weakness, 1 focal finger flexor weakness, 11 elbow flexor weakness, and 2 pain), none of which were considered to be severe or disabling. The mean duration of side effects was 36 days (range 7–120). 

At the end of the study period (1st January 2018), 41 (45.1%) patients were still receiving onaBoNT-A injections. The remaining 50 (54.9%) patients were no longer receiving injections in our clinic: 5 (5.5%) were lost to follow-up, 14 (15.4%) were dissatisfied and discontinued treatment usually after only 2–3 injections, and 4 (4.4%) transitioned to alternative therapy; the reason for discontinuation was financial or unknown in 24 (26.4%) and 3 (3.3%) died of unrelated causes. Additional 30 patients (ET, *n* = 14; dystonia, *n* = 10; other, *n* = 6) did not meet our inclusion criteria as they discontinued onaBoNT-A injections after just 1 visit, either for unknown reasons (*n* = 15), lack of benefit (*n* = 10), adverse effects (*n* = 2), or other reasons (*n* = 3). 

## 3. Discussion

In this study we provide longitudinal, follow-up, data for 91 patients treated with onaBoNT-A for hand tremor over an average period of 2.5 years. To our knowledge, this is the longest duration of follow-up data reported regarding the use of BoNT for hand tremor, although we previously reported our long-term experience with BoNT in the treatment of dystonia over a period of more than 20 years [19]. The mean age of our patient population and the male predominance is similar to those reported in recently published studies of BoNT in the treatment of tremor [20,21,22,23,24,25]. There was a relatively long period of time between onset of symptoms and first injection (mean 23.2 years, up to 75 years). In contrast to our experience with BoNT for dystonia [19], we found no significant difference in the outcome measures (peak effect, global effect, latency of response, or duration of response) between the first and last visit. We found no difference in response to BoNT injections based on underlying etiology of the tremor as measured by the peak effect and global rating. There was, however, a modest but significant increase in the average dose of onaBoNT-A, from 65.0 U to 78.6 U (difference of 13.6 U; *p* = 0.002). The most plausible reason for the 21% increase in average BoNT dose per limb between the first and last visit is that the initial dose is often quite conservative and relatively lower than the estimated maintenance dose to minimize potential adverse effects such as hand weakness. We have observed sustained benefit during the follow-up period (mean, 29.6 months; range, 3–88 months) with a significant proportion of patients reporting moderate or marked improvement of tremor severity and function (score of 3 or 4) on the peak effect rating scale (80.2% versus 85.7%) and global effect rating scale (74.7% versus 80.2%) at the first and last visit. Of the 50 (54.9%) patients who had discontinued treatment with BoNT injections during the study period, 32 (35.2%) did so for unknown or unavoidable reasons, while only 14 (15.4%) were dissatisfied with treatment. 

**Figure 2:**
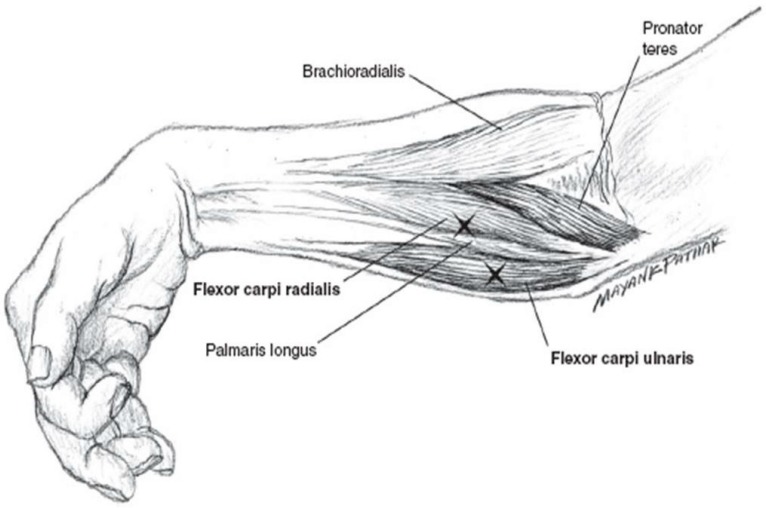
Localization of the forearm flexors most commonly injected in hand tremor. Reprinted with permission from [29], Copyright Cambridge University Press, 2003.

In the earliest study of onaBoNT-A in the treatment of tremor from our center, 10 patients with hand tremor (mixed etiology) received a mean dose of 95 ± 38 U predominantly divided between the forearm flexor and forearm extensor compartment [26]. The peak effect hand tremor was lower (2.0 ± 1.7 versus 3.2 ± 1.3) and the rate of focal weakness was higher (40.0% versus 12.1%) compared to the data reported in the present study. This is consistent with data from the first two double-blind, placebo-controlled trials of BoNT in the treatment of ET [27,28]. In both studies, a pre-determined set of muscles (forearm flexors and extensors) was injected with a fixed dose of BoNT, yielding clinically significant improvement of tremor, however with dose-dependent hand and finger weakness in up to 92% of patients. Because of the high frequency of extensor finger weakness we have since changed our injection technique to favor the forearm flexors while largely avoiding the forearm extensors, thereby reducing the frequency of clinically significant (extensor) wrist and finger weakness while maintaining tremor reduction (Figure 2) [29].

Several well-designed studies have evaluated the use of kinematic tremor analysis coupled with EMG-guidance in the application of BoNT [20,21,23,30,31,32]. Kinematic analysis allows the clinician to objectively characterize tremor at the wrist, elbow and shoulder joint, thereby creating an individual “tremor profile” which can be re-assessed at future visits thus allowing for optimization of the injection pattern. In a study involving 30 patients with ET who were randomized to receive a single injection of either incoBoNT-A (*n* = 19) or placebo (*n* = 11) using a kinematic analysis-based approach the mean total dose of incoBoNT-A was 116.3 ± 53.0 U distributed between multiple muscle compartments [23]. There was a significant reduction in wrist tremor amplitude and in the Fahn-Tolosa-Marin (FTM) tremor rating scale part B at week 4 and 8 post-injection. The injections were associated with approximately 15–20% reduced grip strength in the treatment group at week 4 and finger extensor weakness in 2 (10.5%) patients. There was no long-term data provided in this study but the same investigators conducted an open-label, long-term (96 weeks) study of kinematic analysis-guided incoBoNT-A injections for ET (*n* = 24) and PD (*n* = 28) upper limb tremor [32]. In this study, a mean of 9.4 ± 2.3 versus 10.2 ± 2.6 muscles were injected with a mean of 180.3 ± 74.8 versus 188.5 ± 78.1 U incoBoNT-A in PD and ET, respectively, at the time of the last injection visit. There was a statistically significant reduction of rest and action tremor on items 20 and 21 of the Unified Parkinson’s Disease Rating Scale (UPDRS), FTM tremor rating scale part A–C, and tremor amplitude. A total of 6 patients (11.5% [4 PD and 2 ET]) withdrew from the study due to disabling weakness and another 5 patients (9.6% [3 PD and 2 ET]) withdrew due to lack of benefit. While the rate of significant weakness fluctuated throughout the study, 5.0–29.4% of PD and ET patients experienced significant finger extensor weakness after each injection session. 

In a double-blind, placebo-controlled, cross-over study of 30 patients with PD-related rest and action tremor, Mittal and colleagues injected incoBoNT-A with EMG guidance to identify muscles with tremor activity [22]. IncoBoNT-A or placebo (saline) was injected at the start of the trial with cross-over to the opposite treatment arm at week 12; outcome was assessed at week 4 and 8 after each injection. A mean of 9 (range 7–12) injections into forearm and hand muscles with a mean of 100 U (range, 85–110) incoBoNT-A per patient per visit were performed. IncoBoNT-A injections were associated with significant reduction of tremor affecting activities of daily living as well as rest and action tremor (UPDRS items 16, 20, and 21) with low rates of non-disabling (10%) and disabling (6.6%) hand weakness. Using a similar study design, the same authors performed incoBoNT-A injections in 28 patients (of 33 enrolled) with hand ET [25]. IncoBoNT-A were injected into a mean of 9 (range, 8–14) hand and forearm muscles (80–120 U). IncoBoNT-A treatment led to significant reduction of tremor (FTM tremor rating scale part B and the National Institute of Health Collaborative Genetic Criteria tremor score severity) at weeks 4 and 8 (*p* < 0.05). IncoBoNT-A treatments were safe and well-tolerated, although mild hand weakness occurred in 21% and 1 patient withdrew from the study due to disabling hand weakness. 

Although EMG, ultrasound, and kinematic-guided analysis can be utilized to successfully treat hand tremor with BoNT, no muscle targeting technique has yet been proven to be superior [33,34]. The results described in our study, using palpation and surface anatomy, seem similar to those obtained using kinematic analysis and/or EMG-guided injections [24,27,32,35]. Thus, it is unclear if a technology-guided approach is necessary and whether it yields better outcomes. Indeed, 21.2% of 52 patients treated using kinematic analysis and EMG-guided injections by Samotus et al. [32] withdrew because of adverse effects or lack of benefit, compared to only 15.4% of our 91 patients, although the two populations are not comparable. Technology-guided injections are useful in some instances, such as when the target muscles are difficult to identify, however, it is nearly impossible to sample all 20 forearm muscles plus other, more proximal, muscles to identify all the muscles that contribute to the tremor and target them for BoNT. Even when EMG is used to target the identified muscle the precise site of the tip of the electrode is impossible to validate with any certainty (even with ultrasound) and it is likely that the effects of BoNT will diffuse beyond the boundaries of the intended target [17]. Furthermore, EMG may sometimes be misleading, particularly in patients injected for dystonia, as it may not differentiate between the primary (agonist) and the compensatory (antagonist) muscle contraction, potentially resulting in injection of the wrong muscle. In this regard, evaluation for mirror movements may be helpful [36], particularly in patients with dystonic tremor. Finally, and perhaps most importantly, technology-guided injections are more time-consuming, more painful and more costly [37] without meaningfully improving the outcomes [38]. 

In our longitudinal study we demonstrated that a comparatively low dose of onaBoNT-A injected into a very limited number of muscles (forearm flexors were injected exclusively in 81.3% of patients) guided by palpation and surface anatomy produced clinically meaningful and sustained improvement of tremor in the majority of patients with low rates of non-disabling focal weakness (12.1%). Although we mainly injected the forearm flexors, the injection pattern should always be individualized and optimized based on the characteristics of the patient’s tremor. For an instance, injection of the biceps brachii or pronator teres muscle may be considered in the treatment of the classical supination/pronation tremor in PD [39]. 

The main strength of our report is that it provides a “real world” account of long-term experience with the use of BoNT in hand tremor using a pragmatic, clinical approach. However, we acknowledge that our study has some limitations, particularly due to its open-label and retrospective design. Further, of 30 patients who discontinued treatment with BoNT after only 1 session, 12 (40%) did so due to either troublesome side effects or lack of benefit. We would not, however, consider these as “failures” since 2 or more treatment visits are often required to optimize the outcome. We do not think that bias and placebo response account for the reported outcomes as patients had sustained improvement over time, noticed wearing off between doses, and returned repeatedly for injections, many traveling long distances. Furthermore, it is important to point out that nearly all our patients were previously treated with optimal medical therapies but because of poor response or undesirable side effects were selected for treatment with BoNT. Prospective studies comparing different methods of muscle selection, localization, and injection techniques may provide further validation of our results.

## 4. Conclusions

In conclusion, onaBoNT-A injections are safe and associated with clinically meaningful and sustained improvement of hand tremor in a mixed population of patients with medically refractory hand tremor. 

## 5. Materials and Methods 

### 5.1. Study Description

We conducted a retrospective analysis of our medical records of patients treated with onaBoNT-A injections for hand tremor in the Parkinson’s Disease Center and Movement Disorders Clinic (PDCMDC) at Baylor College of Medicine (BCM). The study was approved by the BCM Institutional Review Board (IRB), number H-42714, approved January 31st, 2018. 

### 5.2. Data Collection 

Patients treated with BoNT for tremor were identified through review of the electronic medical record (EMR) at BCM. The medical history, past treatments, examination and the indication for BoNT treatment was determined by review of the medical record. The “Botulinum Toxin Data Form” (“BoNT Data Form”), which captures injection data (muscle selection, dose, type of BoNT, and response to prior injection) was completed at the initial and all subsequent injection visits. The “peak effect”, one of the two main outcome measures, was defined as the maximum improvement of tremor following BoNT injection, and was assessed by a 0–4 rating scale (0 = no effect; 1 = mild effect, but no functional improvement; 2 = moderate improvement, but no change in functional disability; 3 = moderate change in both severity and function; and 4 = marked improvement in severity and function). The “global rating”, the other outcome measure, was defined as the peak effect score minus 1 point for a mild side effect, such as transient pain or mild weakness, and minus 2 points for a disabling side effect, such as marked weakness causing impairment of function. The “latency of response” was the time from injection of BoNT until the first noticeable response (in days), “duration of maximum response” was the period of time during which the patient experienced peak effect (in weeks), and “total duration of response” represented the time from the first noticeable response to complete wearing off (in weeks). The type, severity, and duration (in days) of side effects were also recorded. 

### 5.3. Selection Criteria

Inclusion criteria for this study were: (1). Presence of troublesome hand tremor that is refractory to medical therapy, including optimized trial of propranolol, primidone, topiramate for ET; levodopa, dopamine agonists or anticholinergics for PD tremor; and anticholinergics for dystonic tremor, (2) chemodenervation using onaBoNT-A injections of the arm(s) for hand tremor, (3) minimum of two BoNT injection visits between 1st January 2010 (introduction of EMR at BCM), and 1st January 2018 with follow-up data, (4) follow-up within 1 year after the first and last injection, and (5) age older than 18 years at the time of the first injection. Patients first treated prior to introduction of the EMR were included in this study, but their “initial visit” was recorded as their first BoNT injection visit after introduction of the EMR. Injection and outcome data were analyzed for the first and last visit while information regarding side effects (type and duration) were captured for all visits. This approach was chosen for simplicity since some patients had more than a dozen injection visits during the study period which would make it difficult to analyze all outcomes at each visit separately. We further recorded the reason for discontinuation of onaBoNT-A injections (if known). 

### 5.4. Botulinum Toxin Injection

We individualized the target muscle and dosage based on the amplitude and character of the tremor, starting at a relatively low dose and usually optimizing the treatment over subsequent 2–3 visits. The majority of patients received an initial injection of approximately 25–75 units (U) of onaBoNT-A per limb divided between flexor carpi radialis (2/3) and flexor carpi ulnaris (1/3) (Figure 2). We do not routinely utilize electromyography (EMG) or ultrasound, but rely on palpation and surface anatomy for guidance in most BoNT injections. 

### 5.5. Statistical Analysis

Using descriptive statistics, we analyzed the following information: age at first injection visit, disease duration, number of injection visits, duration of treatment with onaBoNT-A, average dose of onaBoNT-A per limb, peak effect, global rating, latency of response, duration of maximum response, and total duration of response. A paired *t*-test was used to compare continuous data outcomes (e.g., BoNT dose) at the first and last injection visit. Ordinal outcomes (e.g., peak effect) were compared using nonparametric tests.

## Figures and Tables

**Table 1:** Botulinum toxin in hand tremor: Demographics and baseline data (*n* = 91).

	Mean	SD	Range
Age at first injection (years)	64.8	12.8	18–93
Tremor duration at first injection (years)	23.2	17.5	0.5–75
Follow-up period (months)	29.6	25.1	3–88
Number of onaBoNT-A sessions	7.7	6.3	2–31
OnaBoNT-A units per session ^†^	71.8	37.3	22.5–225
OnaBoNT-A units ^†^ (per treatment indication)			
ET (*n* = 53)	71.3	36.7	22.5–225
PD (*n* = 6)	47.9	11.5	37.5–67.5
Dystonia (*n* = 31)	77.3 *	41.0	27.5–187.5
COT (*n* = 1)	70	-	-

SD = Standard deviation, ET = Essential tremor, PD = Parkinson’s disease, onaBoNT-A = onabotulinumtoxin-A, COT = Cerebellar outflow tremor. * *p* = 0.49 for comparison with ET. ^†^ mean dose per limb (first and last visit combined).

**Table 2:** Injection strategy at the last visit according to tremor etiology (*n* = 91).

Treatment Indication (Muscles Injected/Limbs Injected)					
	ET	PD	Dystonia	COT	Total
Deltoid	0/99	0/6	1/37	1/1	2/143
Biceps	10/99	0/6	3/37	1/1	14/143
Triceps	1/99	0/6	0/37	0/1	1/143
Pronator teres	4/99	0/6	5/37	0/1	9/143
FCU	94/99	6/6	34/37	1/1	135/143
FCR	92/99	6/6	26/37	1/1	125/143
FDS	3/99	0/6	4/37	0/1	7/143
ADM	0/99	0/6	1/37	0/1	1/143
APB	1/99	0/6	11/37	0/1	12/143
ED	0/99	0/6	1/37	0/1	1/143
EPB	0/99	0/6	1/37	0/1	1/143
UNS extensor	0/99	0/6	1/37	0/1	1/143

ET = Essential tremor, PD = Parkinson’s disease, COT = Cerebellar outflow tremor, FCU = Flexor carpi ulnaris, FCR = Flexor carpi radialis, FDS = Flexor digitorum superficialis, ADM = Abductor digiti minimi, APB = Abductor pollicis brevis, ED = extensor digitorum, EPB = Extensor pollicis brevis, UNS extensor = unspecified extensor muscle.

**Table 3:** Outcomes following onaBoNT-A injections for hand tremor (*n* = 91).

	First Injection (*n*)	Last Injection (*n*)	*p* *
Mean onaBoNT-A units	65.0 ± 31.2 (91)	78.6 ± 51.1 (91)	0.002
Mean global rating	3.0 ± 1.3 (91)	3.2 ± 1.2 (91)	0.06
Mean peak effect	3.2 ± 1.3 (91)	3.4 ± 1.1 (91)	0.18
Moderate or marked benefit (score of 3 or 4)			
Global rating	74.7%	80.2%	-
Peak effect	80.2%	85.7%	-
Mean latency of response, days	4.5 ± 4.3 (81)	3.8 ± 3.0 (78)	0.10
Mean total duration of response, weeks ^†^	12.7 ± 3.6 (46)	12.8 ± 2.8 (37)	0.87

OnaBoNT-A = onabotulinumtoxin-A. * *p* < 0.05 indicating a statistically significant result. ^†^ Data not included for patients with persistent response (27 versus 41) or lack of data (18 versus 13) at the time of follow-up after the first and last injection.

## References

[1] Bhatia K.P., Bain P., Bajaj N., Elble R.J., Hallett M., Louis E.D., Raethjen J., Stamelou M., Testa C.M., Deuschl G. (2018). Consensus Statement on the classification of tremors. From the task force on tremor of the International Parkinson and Movement Disorder Society. Mov. Disord..

[2] Louis E.D., Machado D.G. (2015). Tremor-related quality of life: A comparison of essential tremor vs. Parkinson’s disease patients. Park. Relat. Disord..

[3] Louis E.D., Ferreira J.J. (2010). How common is the most common adult movement disorder? Update on the worldwide prevalence of essential tremor. Mov. Disord..

[4] Pringsheim T., Jette N., Frolkis A., Steeves T.D. (2014). The prevalence of Parkinson’s disease: A systematic review and meta-analysis. Mov. Disord..

[5] Thenganatt M.A., Jankovic J. (2016). The relationship between essential tremor and Parkinson’s disease. Park. Relat. Disord..

[6] Dirkx M.F., Zach H., Bloem B.R., Hallett M., Helmich R.C. (2018). The nature of postural tremor in Parkinson disease. Neurology.

[7] Jankovic J. (2016). How Do I Examine for Re-Emergent Tremor?. Mov. Disord. Clin. Pract..

[8] Fasano A., Bove F., Lang A.E. (2014). The treatment of dystonic tremor: A systematic review. J. Neurol. Neurosurg. Psychiatry.

[9] Pandey S., Sarma N. (2016). Tremor in dystonia. Park. Relat. Disord..

[10] Jiménez M.C., Vingerhoets F.J.G. (2012). Tremor revisited: Treatment of PD tremor. Park. Relat. Disord..

[11] Schneider S.A., Deuschl G. (2014). The treatment of tremor. Neurotherapeutics.

[12] Buhmann C., Huckhagel T., Engel K., Gulberti A., Hidding U., Poetter-Nerger M., Goerendt I., Ludewig P., Braass H., Choe C.U. (2017). Adverse events in deep brain stimulation: A retrospective long-term analysis of neurological, psychiatric and other occurrences. PLoS ONE.

[13] Baizabal-Carvallo J.F., Jankovic J. (2016). Movement disorders induced by deep brain stimulation. Park. Relat. Disord..

[14] Schreglmann S.R., Krauss J.K., Chang J.W., Martin E., Werner B., Bauer R., Hägele-Link S., Bhatia K.P., Kägi G. (2018). Functional lesional neurosurgery for tremor: Back to the future?. J. Neurol. Neurosurg. Psychiatry.

[15] Elble R.J., Shih L., Cozzens J.W. (2018). Surgical treatments for essential tremor. Expert Rev. Neurother..

[16] Mohammed N., Patra D., Nanda A. (2018). A meta-analysis of outcomes and complications of magnetic resonance-guided focused ultrasound in the treatment of essential tremor. Neurosurg. Focus.

[17] Ramirez-Castaneda J., Jankovic J., Comella C., Dashtipour K., Fernandez H.H., Mari Z. (2013). Diffusion, spread, and migration of botulinum toxin. Mov. Disord..

[18] Jankovic J. (2018). An update on new and unique uses of botulinum toxin in movement disorders. Toxicon.

[19] Ramirez-Castaneda J., Jankovic J. (2014). Long-term efficacy, safety, and side effect profile of botulinum toxin in dystonia: A 20-year follow-up. Toxicon.

[20] Samotus O., Rahimi F., Lee J., Jog M. (2016). Functional ability improved in essential tremor by incobotulinumtoxinA injections using kinematically determined biomechanical patterns—A new future. PLoS ONE.

[21] Rahimi F., Samotus O., Lee J., Jog M. (2015). Effective management of upper limb parkinsonian tremor by incobotulinumtoxinA injections using sensor-based biomechanical patterns. Tremor Other Hyperkinet. Mov..

[22] Mittal S.O., Machado D., Richardson D., Dubey D., Jabbari B. (2017). Botulinum toxin in Parkinson disease tremor: A randomized, double-blind, placebo-controlled study with a customized injection approach. Mayo Clin. Proc..

[23] Jog M., Lee J., Althaus M., Scheschonka A., Dersch H., Simpson D.M., ET Study Team (2017). Efficacy and safety of incobotulinumtoxinA (inco/A) for essential tremor using kinematics-guided clinical decision support: A randomized, double-blind, placebo-controlled trial. Mov. Disord..

[24] Kim S.D., Yiannikas C., Mahant N., Vucic S., Fung V.S. (2014). Treatment of proximal upper limb tremor with botulinum toxin therapy. Mov. Disord..

[25] Mittal S.O., Machado D., Richardson D., Dubey D., Jabbari B. (2018). Botulinum toxin in essential hand tremor—A randomized double-blind placebo-controlled study with customized injection approach. Park. Relat. Disord..

[26] Jankovic J., Schwartz K.S. (1991). Botulinum toxin treatment of tremors. Neurology.

[27] Jankovic J., Schwartz K., Clemence W., Aswad A., Mordaunt J. (1996). A randomized, double-blind, placebo-controlled study to evaluate botulinum toxin type A in essential hand tremor. Mov. Disord..

[28] Brin M.F., Lyons K.E., Doucette J., Adler C.H., Caviness J.N., Comella C.L., Dubinsky R.M., Friedman J.H., Manyam B.V., Matsumoto J.Y. (2001). A randomized, double masked, controlled trial of botulinum toxin type A in essential hand tremor. Neurology.

[29] Jankovic J. (2013). The use of botulinum toxin in tic disorders and essential hand and head tremor. Manual of Botulinum Toxin Therapy.

[30] Rahimi F., Bee C., Debicki D., Roberts A.C., Bapat P., Jog M. (2013). Effectiveness of BoNT A in Parkinson’s disease upper limb tremor management. Can. J. Neurol. Sci..

[31] Samotus O., Kumar N., Rizek P., Jog M. (2018). Botulinum toxin type A injections as monotherapy for upper limb essential tremor using kinematics. Can. J. Neurol. Sci..

[32] Samotus O., Lee J., Jog M. (2017). Long-term tremor therapy for Parkinson and essential tremor with sensor-guided botulinum toxin type A injections. PLoS ONE.

[33] Zakin E., Simpson D. (2017). Botulinum toxin in management of limb tremor. Toxins.

[34] Karp B., Alter K. (2017). Muscle selection for focal limb dystonia. Toxins.

[35] Pacchetti C., Mancini F., Bulgheroni M., Zangaglia R., Cristina S., Sandrini G., Nappi G. (2000). Botulinum toxin treatment for functional disability induced by essential tremor. Neurol. Sci..

[36] Sitburana O., Wu L.J., Sheffield J.K., Davidson A., Jankovic J. (2009). Motor overflow and mirror dystonia. Park. Relat. Disord..

[37] Wu C., Xue F., Chang W., Lian Y., Zheng Y., Xie N., Zhang L., Chen C. (2016). Botulinum toxin type A with or without needle electromyographic guidance in patients with cervical dystonia. Springerplus.

[38] Jankovic J. (2001). Needle EMG guidance for injection of botulinum toxin. Needle EMG guidance is rarely required. Muscle Nerve.

[39] Sheffield J.K., Jankovic J. (2007). Botulinum toxin in the treatment of tremors, dystonias, sialorrhea and other symptoms associated with Parkinson’s disease. Expert Rev. Neurother..

